# Cross-Cultural Adaptation and Validation of the Italian Version of the Dysphagia Handicap Index (I-DHI)

**DOI:** 10.1007/s00455-021-10369-2

**Published:** 2021-09-28

**Authors:** Daniela Ginocchio, Aurora Ninfa, Nicole Pizzorni, Christian Lunetta, Valeria Ada Sansone, Antonio Schindler

**Affiliations:** 1 NEuroMuscular Omnicentre, Fondazione Serena Onlus, Milan, Italy; 2 Department of Biomedical and Clinical Sciences “Luigi Sacco”, Università degli Studi di Milano, Via GB Grassi 74, 20154 Milan, Italy; 3 Department of Pathophysiology and Transplantation, Università degli Studi di Milano, Via Francesco Sforza 35, 20122 Milan, Italy; 4 Istituti Clinici Scientifici Maugeri IRCCS, Milan, Italy; 5 NEMO Lab, Milan, Italy; 6 Neuroriabilitation Unit, Department of Biomedical Sciences for Health, Università degli Studi di Milano, Via Mangiagalli 31, 20133 Milan, Italy; 7 Phoniatric Unit, Department of Biomedical and Clinical Sciences “L. Sacco”, Università Milano, Via GB Grassi 74, 20154 Milan, Italy

**Keywords:** Deglutition disorders, Dysphagia, Health-related Quality of Life, Self-assessment, Fiberoptic Endoscopic Evaluation of Swallowing

## Abstract

The Dysphagia Handicap Index (DHI) is a valid Health-related Quality of Life (HRQOL) questionnaire for patients with oropharyngeal dysphagia (OD) of heterogeneous etiologies. The study aimed at crossculturally translating and adapting the DHI into Italian (I-DHI) and analyzing I-DHI reliability, validity, and interpretability. The I-DHI was developed according to Beaton et al. 5-stage process and completed by 75 adult OD patients and 166 healthy adults. Twenty-six patients filled out the I-DHI twice, 2 weeks apart, for test–retest reliability purposes. Sixty-two patients completed the Italian-Swallowing Quality of Life Questionnaire (I-SWAL-QoL) for criterion validity analysis. Construct validity was tested comparing I-DHI scores among patients with different instrumentally assessed and self-rated OD severity, comparing patients and healthy participants and testing Spearman’s correlations among I-DHI subscales. I-DHI interpretability was assessed and normative data were generated. Participants autonomously completed the I-DHI in maximum 10 min. Reliability proved satisfactory for all I-DHI subscales (internal consistency: *α* > .76; test–retest reliability: intraclass correlation coefficient > .96, *k* = .81). Mild to moderate correlations (− .26 ≤ *ρ* ≤ − .72) were found between I-DHI and I-SWAL-QoL subscales. Construct validity proved satisfactory as (i) moderate to strong correlations (.51 ≤ *ρ* ≤ .90) were found among I-DHI subscales; (ii) patients with more severe instrumentally or self-assessed OD reported higher I-DHI scores (*p* < .05); and (iii) OD patients scored higher at I-DHI compared to healthy participants (*p* < .05). Interpretability analyses revealed a floor effect for the Emotional subscale only and higher I-DHI scores (*p* < .05) for healthy participants > 65 years. In conclusion, the I-DHI is a reliable and valid HRQOL tool for Italian adults with OD.

## Introduction

Oropharyngeal dysphagia (OD) is defined as any structural or functional abnormality in oropharyngeal swallowing physiology as a result of heterogeneous clinical conditions, such as Head and Neck Cancer (HNC) and respiratory, systemic, or neurological diseases [1]. OD complications include aspiration pneumonia, malnutrition, and dehydration [2]. OD may be experienced by patients as discomfort on swallowing food and liquids [3]. Besides affecting physical health, OD impacts patients’ daily activities and social life [4]. Increased OD severity is associated with decreased Health-related quality of life (HRQOL) [5]. HRQOL is a broad subjective construct that can be defined as the individuals’ perception of the impact of their health status, illness, and treatment on their physical, emotional, mental, and social functioning [6].

Clinician-driven measures are commonly used to assess patients’ swallowing function [1]. Videofluoroscopic Swallowing Study (VFSS) and Fiberoptic Endoscopic Evaluation of Swallowing (FEES) are well-established gold standards for the instrumental assessment of pathological swallowing patterns underlying OD [7], alongside clinical assessment with swallowing trials [1]. In recent years, the investigation of patients’ perspective through Patient-Reported Outcome (PRO) tools has gained increasing importance, since it may not correlate with clinician-driven measures [8]. HRQOL is usually investigated by means of PRO tools due to the subjective nature of the construct.

There are several HRQOL measures specifically designed for patients with OD [9]. Nevertheless, Timmerman et al. [9] advocated the development of PRO tools with sound psychometric properties in order to generate proper conclusions and enhance the methodological quality of research studies in the OD field.

Among HRQOL measures, the Swallowing Quality of Life Questionnaire (SWAL-QoL) [10] and the Dysphagia Handicap Index (DHI) [11] were established to have the most satisfactory psychometric properties [9]. The SWAL-QoL, the MD Anderson Dysphagia Inventory (MDADI) [12], and the Eating Assessment Tool (EAT-10) [13] were adapted and validated into Italian [14–16], although presenting some caveats. In fact, SWAL-QoL probe statements tend to be abstract, with complex wording and different response choices among subscales, raising comprehension concerns [11]. Moreover, using Rasch Analysis, Cordier et al. [17] highlighted concerns relating to the number of response options for each item, the number of items for each subscale, and the need to further revise the questionnaire. The MDADI is a widely used HRQOL tool but only applicable to head and neck cancer patients. Finally, the EAT-10 aims to assess OD-related functional health status (i.e., the symptomatic severity of dysphagia as experienced by the patient [6]) rather than HRQOL. EAT-10 was also reported to have weak psychometric properties [18].

The DHI was designed as a clinically efficient, easy to complete HRQOL questionnaire for individuals with OD deriving from a variety of medical diagnoses. With 25 items, the DHI investigates the physical, functional, and emotional impacts that OD may have on the patient’s life and provides a self-evaluation of OD severity. To date, the DHI has been used to distinguish between different levels of HRQOL in adult OD patients of etiological heterogeneity in crosssectionally designed studies [19–24]. Moreover, the DHI was used to assess changes in swallowing self-perception as an outcome measure of treatment efficacy in patients with Parkinson’s disease [25, 26], cricopharyngeal spasm and pharyngeal pouch [27], post-thyroidectomy dysphagia [28], and Head and Neck Cancer [29]. The DHI has attracted growing attention in the international literature, presenting overall satisfactory psychometric properties [9]. Besides its original English version, it has been crossculturally validated into Arabic [30], Persian [31, 32], Japanese [33], Hebrew [34], Kannada [35], and Korean [36].

As a validated Italian version of the DHI was not available, the present study aimed at (i) translating and crossculturally adapting the DHI into Italian and naming it the Italian-DHI (I-DHI), (ii) assessing I-DHI psychometric properties in terms of reliability (internal consistency, test–retest reliability) and validity (criterion validity and construct validity), and (iii) assessing the interpretability of the developed tool. The validation of the Italian version of the DHI may offer a common language to crossculturally compare clinical and research results gathered with the same instrument, namely the DHI. It may allow the comparison of Italian results with the ones of non-Italian-speaking countries. In addition, the I-DHI could be used to investigate HRQOL of patients with OD in Italian-speaking countries (i.e., Switzerland, San Marino, and Vatican City) and Italian-speaking communities across countries (e.g., Albania, Argentina, Australia, Belgium, Bosnia and Herzegovina, Brazil, Canada, Costa Rica, Croatia, Malta, Egypt, Eritrea, France, Germany, Israel, Libya, Liechtenstein, Luxembourg, Paraguay, the Philippines, Puerto Rico, Romania, Saudi Arabia, Slovenia, Tunisia, the United Arab Emirates, the UK, the USA, and Venezuela).

According to the previous DHI validation studies, it was hypothesized that the I-DHI would demonstrate satisfactory psychometric properties and interpretability.

## Materials and Methods

### Study Design

This cross-sectional non-randomized prospective study with controls was carried out according to the Declaration of Helsinki and previously approved by the local Institutional Review Board. Written informed consent was obtained for all the participants included in the study. Authors of the original English version of the DHI consented to the completion of the present study.

Four main phases were followed: I-DHI development (phase 1), which encompassed the translation of items for the DHI cross-cultural adaptation into Italian, I-DHI reliability analysis (phase 2), I-DHI validity analysis (phase 3), and I-DHI interpretability analysis (phase 4). The COnsensus-based Standards for the selection of health Measurement INstruments (COSMIN) [37] checklist was followed as a reference for the completion of the present study.

### Participants

Participants were selected among the consecutive cohort of patients who were referred for known or suspected OD to two Phoniatrics clinics in Northen Italy between November 2019 and February 2020 and between June and November 2016. In order to examine clinical validity, healthy participants were randomly selected from the community. Inclusion criteria were (i) presence of OD deriving from any etiology, in patients; (ii) no history of OD, related diseases, or feeding tube placement, in healthy participants; (iii) age ≥ 18 years; and (iv) ability to independently read a written text. Both patients and healthy participants were excluded from the study if they presented a state of cognitive decline (i.e., Mini Mental State Examination [38] < 24).

### Procedures

Instrumental, clinical, and demographic data were recorded and stored anonymously on the institutional computer’s hard drive, while backup copies were saved on external memory drives. Patients completed the I-DHI and underwent a FEES examination for the assessment of OD in the same session. FEES was performed using a XION EF-N flexible endoscope with a 3.4 mm diameter and a length of 320 mm (XION GmbH, Berlin, Germany) mounted on an EndoSTROBE camera (XION GmbH, Berlin, Germany). Two Phoniatricians with > 20 years of clinical and research experience in dysphagia and FEES conduction carried out the FEES examinations. FEES examinations were conducted using thin liquid (5–10–20 ml of blue-dyed water × 3 trials for each volume; International Dysphagia Diet Standardization Initiative [39]—IDDSI 0; Viscosity: < 50 mPa·s at 50 s^−1^ and 300 s^−1^), pureed food (5–10–20 ml of pudding × 3 trials for each volume; IDDSI 4; Viscosity: 2583.3 ± 10.41 mPa·s at 50 s^−1^ and 697.87 ± 7.84 mPa·s at 300 s^−1^), and regular food (half biscuit × 2 trials; IDDSI 7 Regular). In the present study, the viscosity levels of liquids and pureed food complied with the descriptors of the National Dysphagia Diet Task Force [40]. All the patients followed the same bolus administration order of liquids (3 × 5 ml, 3 × 10 ml, 3 × 20 ml), puree (3 × 5 ml, 3 × 10 ml, 3 × 20 ml), and solids. If either consistency or volume was considered unsafe to be administered or if severe swallowing efficacy impairment was observed, the FEES protocol was not completed with unsafe volumes/consistencies. Owing to safety reasons, the FEES protocol was interrupted if at least one of the following conditions occurred: (i) laryngospasm during the water test in the clinical assessment; (ii) severe impairment of the oral control of the bolus with pureed food which led to chocking; (iii) and severe impairment of the oral preparatory swallowing stage with solids which prevented the processing of the solid into a bolus. Based on FEES video recordings, OD severity was rated with the Dysphagia Outcome and Severity Scale (DOSS) [41]. The DOSS is a 7-point ordinal scale with scores 1–2 indicating severe and moderately severe OD with non-oral nutrition necessary, scores from 3 to 5 corresponding to moderate, mild moderate, and mild OD with a modified diet on a full per-oral nutrition, and scores 6–7 representing functional and normal swallowing abilities with a normal diet. In addition, swallowing safety was assessed using the Penetration-aspiration scale (PAS) [42, 43]. PAS is an 8-point ordinal scale, with score 1 indicating no penetration and aspiration, score 2 representing transient penetration with ejection, scores 3–5 laryngeal penetration without ejection and/or reaching the vocal folds, and scores 6–8 tracheal aspiration. In accord with recent research studies addressing PAS psychometric properties [44, 45], PAS scores 1–2 were considered to reflect normal swallowing function. Penetration was scored as present with PAS > 2 ≤ 5 [44, 45], while aspiration with PAS > 5 [42]. A PAS score was attributed to each administered bolus. The worst bolus (i.e., the bolus with highest PAS score) for each consistency tested and the worst PAS score among all consistencies was considered for analysis purposes. As appropriate for ordinal measures [46], inter-rater agreement was calculated using the linear weighted kappa coefficient for both DOSS and PAS scales.

### Phase 1: I-DHI Development

The original version of the DHI [11] was translated and culturally adapted into Italian, following the 5-stage process described by Beaton et al. [47]. Items of the original DHI questionnaire were independently translated into Italian by a bilingual expert panel composed of two speech and language pathologists and two Phoniatricians familiar with OD patients care with a conceptual translation being preferred over a literal one (stage I: forward translation). An Italian final consensus version was obtained after addressing idiomatic, semantic, and conceptual issues (stage II: synthesis). Three independent professional translators who had no knowledge of the questionnaire translated it back into English (stage III: back translation). Subsequently, conceptual and cultural equivalence of all reports were reviewed by the three translators and the expert panel and a final version of the I-DHI was produced (stage IV: expert committee review). The final version of the I-DHI was pilot-tested on 15 consenting patients with OD who were fluent in both Italian and English and approved, since no major suggestions emerged (stage V: pretesting).

The questionnaire aimed at investigating OD patients’ HRQOL, in terms of reported OD physical symptoms, consequences of OD on social and psychological functioning, and perceived dysphagia severity. Mirroring DHI original version, the I-DHI aimed to distinguish between different levels of HRQOL in adults with OD of etiological heterogeneity.

The final version of the I-DHI consisted of 25 items divided into three subscales (Appendix): (i) the Physical subscale refers to the person’s self-perception of physical discomfort due to OD (9 items), (ii) the Functional subscale reflects the impact of OD on the person’s daily activities (9 items), and (iii) the Emotional subscale represents the person’s affective response to OD (7 items). Participants were invited to state whether each item applied to them “never,” “some of the time,” or “all the time,” scoring 0, 2, and 4, respectively. A total I-DHI score was obtained summing the scores of the subscales (possible range 0–100), with higher values meaning worse HRQOL. On completion of the questionnaire, participants were requested to rate the perceived severity of their swallowing problem using a 1–7-point ordinal scale with 1 = “no problem,” 4 = “moderate swallowing problem,” and 7 = “severe problem.”

### Phase 2: I-DHI Reliability

The I-DHI internal consistency (i.e., the degree of the interrelatedness among the items) and test–retest reliability (i.e., the proportion of the total variance in the measurements which is due to ‘true’ differences between patients) [37] were examined. Cronbach’s *α* was computed for each I-DHI subscale and total score to analyze internal consistency. In order to assess I-DHI test–retest reliability, a randomly selected subsample of 26 patients completed the I-DHI a second time, 2 weeks after the initial assessment. The period of 2 weeks consented a reduction in recall bias and guaranteed stability of patients’ health status between the two assessment sessions. During this period, patients did not undergo any medical, surgical, or behavioral treatment for swallowing. Participants selected for assessing test–retest reliability were compared to the main sample for age, gender, and diagnosis (categorized as neurological disorders, respiratory disorders, head and neck cancer, and other disorders). Demographic and clinical characteristics of this subsample are presented in Table 1.

**Table 1:** Demographic and clinical characteristics of study populations

Type of study	Study population	Diagnosis (*N*; %)	Age (years)	Gender *N* (%)	
			Mean ± SD (min–max)	M	F
Internal consistency	75 patients with oropharyngeal dysphagia	Amyotrophic lateral sclerosis (40; 53.3%) Head and neck cancer (11; 14.7%) Myotonic dystrophy type 1 (7; 9.3%) Multiple system atrophy (4; 5.4%) Parkinson’s disease/Parkinsonism (3; 4.0%) Stroke/brain hemorrhage (3; 4.0%) Others^a^ (7; 9.3%)	65.4 ± 11.0 (35–85)	43 (57.3%)	32 (42.7%)
Test–retest reliability	26 patients with oropharyngeal dysphagia	Amyotrophic lateral sclerosis (13; 50.0%) Myotonic dystrophy type 1 (3; 11.5%) Parkinson’s disease/Parkinsonism (3; 11.5%) Stroke/brain hemorrhage (2; 7.7%) Others^b^ (5; 19.3%)	66.7 ± 11.0 (42–81)	16 (61.5%)	10 (38.5%)
Criterion validity	62 patients with oropharyngeal dysphagia	Amyotrophic lateral sclerosis (40; 64.5%) Myotonic dystrophy type 1 (6; 9.7%) Multiple system atrophy (4; 6.5%) Parkinson’s disease/Parkinsonism (3; 4.8%) Stroke/brain hemorrhage (3; 4.8%) Others^c^ (6; 9.7%)	65.3 ± 10.9 (35–85)	33 (53.2%)	29 (46.8%)
Construct validity	63 patients with oropharyngeal dysphagia	Amyotrophic lateral sclerosis (34; 54.0%) Head and neck cancer (9; 14.3%) Myotonic dystrophy type 1 (6; 9.5%) Multiple system atrophy (4; 6.3%) Parkinson’s disease/Parkinsonism (3; 4.8%) Stroke/brain hemorrhage (2; 3.2%) Others^d^ (5; 7.9%)	66.0 ± 10.9 (35–85)	37 (58.7%)	26 (41.3%)
Construct validity	166 healthy adults	–	46.7 ± 19.0 (18–92)	79 (47.6%)	87 (52.4%)

^a^Bariatric surgery; Huntington’s disease; respiratory insufficiency; Kennedy’s disease; progressive supranuclear palsy; extrapyramidal syndrome; Guillain–Barré syndrome

^b^Kennedy’s disease; multiple system atrophy; progressive supranuclear palsy; extrapyramidal syndrome; Guillain–Barré syndrome

^c^Bariatric surgery; Huntington’s disease; respiratory insufficiency; Kennedy’s disease; extrapyramidal syndrome; Guillain–Barré syndrome

^d^Bariatric surgery; respiratory insufficiency; Kennedy’s disease; progressive supranuclear palsy; Guillain–Barré syndrome

### Phase 3: I-DHI Validity

I-DHI validity was examined, that is, the degree to which the I-DHI measures the construct(s) it purports to measure (i.e., HRQOL of adults with OD). To this purpose, criterion validity (i.e., the degree to which the scores of an instrument are an adequate reflection of a gold standard) and construct validity (i.e., the degree to which the scores of an instrument are consistent with hypotheses) [37] were tested.

#### I-DHI Criterion Validity

In order to investigate I-DHI criterion validity, the Italian version of the Swallowing Questionnaire (I-SWAL-QoL) [14] was chosen as the gold standard owing to its satisfactory psychometric properties [9] and wide use in clinical practice and research studies. The I-SWAL-QoL consists of 44 items divided into 11 subscales, namely, the burden of eating difficulty, eating duration, eating desire, symptoms, food selection, communication, fear, mental health, social functioning, fatigue, and sleep. Each subscale encompasses a variable number of items, from 2 to 14. Each item is scored on a 5-point Likert scale from 1 to 5, with lower scores indicating worse HRQOL. In order to assess criterion validity, correlation analyses between I-DHI and I-SWAL-QoL subscales were performed. Inverse correlations were expected, as for I-DHI and I-SWAL-QoL a worse HRQOL is suggested by higher and lower scores, respectively. No previous study investigated DHI criterion validity, and thus no more precise hypotheses on correlational strength could be made. A randomly selected subsample of 62 patients with OD completed the SWAL-QoL for criterion validity purposes. The demographic and clinical characteristics of this subsample are presented in Table 1.

#### I-DHI Construct Validity

In order to investigate I-DHI construct validity, the following hypotheses were made: (i) direct moderate (*ρ* = .50–.70) to strong (ρ > .70) correlations were predicted among I-DHI subscales, Total score and self-rated OD severity; (ii) significant inverse correlations were expected between I-DHI and OD severity on FEES scored using the DOSS; (iii) based on Shapira-Galitz et al. findings [34], weak correlations were anticipated between I-DHI and swallowing safety measured with PAS, while significantly higher I-DHI Functional subscale scores were expected for patients with penetration (PAS = 3–5) and/or aspiration (PAS = 6–8) compared to patients with safe swallow; (iv) significantly higher I-DHI subscales and Total scores were anticipated among patients with more severe self-rated OD for all pairwise comparisons (patients were divided into categories based on the I-DHI self-rated OD severity score: 1 = normal swallow, 2–3 = mild swallowing problem, 4–5 = moderate swallowing problem, and 6–7 = severe swallowing problem); (v) as a measure of I-DHI clinical validity, patients with OD were hypothesized to report significantly higher scores for all the I-DHI subscales and the Total score compared to healthy participants.

### Phase 4: I-DHI Interpretability

Interpretability is defined as the attribution of qualitative meaning to an instrument’s quantitative scores or change in scores. It is regarded as an important measurement characteristic, although not encompassed among psychometric properties [37]. In order to describe the features of the score distribution, the number of items, the possible and observed score ranges, the observed number of distinct scores, and the median (Interquartile Range—IQR) for each subscale were presented. Floor and ceiling effects were considered present if at least 15% of participants reported the lowest or the highest possible scores, respectively [48]. Norm values (median; IQR) were presented to establish a baseline distribution for I-DHI scores. For both patients and healthy participants, differences among gender and age groups (18–39, 40–64, ≥ 65 years) were investigated.

### Statistical Analysis

The present study adopted the quality criteria for measurement properties as defined by Terwee et al. [48]. Data were reported as median (IQR) or as absolute (relative) frequencies. Inter-rater agreement for the FEES outcomes was calculated using the linear weighted kappa coefficient. Kappa values were interpreted as poor (0), slight (0.00–0.20), fair (0.21–0.40), moderate (0.41–0.60), substantial (0.61–0.80), and almost perfect (0.81–1) agreement [46]. The normality of the variable distributions was assessed with the Kolmogorov–Smirnov test. Non-parametric tests were conducted since ordinal variables and non-normally distributed continuous variables were analyzed. Internal consistency was considered satisfactory if Cronbach’s *α* values were between 0.70 and 0.90. As suggested by Terwee et al. [48], test–retest reliability was assessed using Intraclass Correlation Coefficient (ICC) two-way random effects model, single measures, absolute agreement for the I-DHI subscales and Total score, and with weighted Cohen’s *k* for the self-rated OD severity subscale. ICC_agreement_ and the weighted Cohen’s *k* values > 0.70 indicated acceptable test–retest reliability. Correlation analyses were performed using Spearman’s correlation coefficient. Correlations were considered mild for values between 0.30 and 0.50, moderate for values between 0.50 and 0.70, and strong for values > 0.70 [49]. For the assessment of criterion validity, correlational strength was considered satisfactory for values > 0.70 [48]. Comparisons between patients and healthy participants and among groups of patients were assessed with the U Mann Whitney (two groups comparisons) or the Kruskal–Wallis tests (multiple groups comparisons). Multiple comparisons were corrected with Bonferroni’s procedure. For all the analyses, a *p*-value smaller than 0.05 was considered significant. All the statistical procedures were carried out with IBM SPSS Statistics 26.0® package for Mac (SPSS Inc., Chicago, IL). Missing values were excluded from the analyses so that for each considered variable, only the available data were analyzed (pairwise deletion).

## Results

### Participants

Seventy-five patients with OD of etiological heterogeneity (M 56.0%; age 65.4 ± 11.0 years) and 166 healthy participants (M 47.6%; age 46.7 ± 19.0 years) were recruited for the present study. Participants’ demographic and clinical characteristics are presented in Table 1.

FEES was performed in 63 patients. On the FEES examination, OD severity rated with DOSS was 3 (3–5) as median (IQR). Four (6.3%) patients had severe OD (DOSS = 1), 6 (9.5%) moderately severe OD (DOSS = 2), 22 (34.9%) moderate OD (DOSS = 3), 12 (19.1%) mild moderate OD (DOSS = 4), 17 (27.0%) mild OD (DOSS = 5), and 2 (3.2%) patients presented a functional swallow (DOSS = 6).

Swallowing safety scored with PAS was 7 (1–8) as median (IQR) with the worst consistency tested, 3 (1–8) with liquids, 1 (1–3) with puree, and 1 (1–2.3) with solids. Regardless of the consistency, 21 (33.3%) patients presented no sign of penetration or aspiration (PAS = 1–2), while 8 (12.7%) showed penetration (PAS = 3–5) and 34 (54.0%) aspiration (PAS = 6–8). Considering the consistencies separately, 30 (47.6%), 38 (60.3%), and 26 (41.3%) patients showed no sign of penetration or aspiration (PAS = 1–2) with liquids, puree, and solids, respectively. Twelve (19.1%), 7 (11.1%), and 2 (3.2%) patients presented penetration (PAS = 3–5) and 21 (33.3%), 8 (12.7%), and 6 (9.5%) aspiration (PAS = 6–8) with liquids, puree, and solids, respectively. For safety reasons, 10 (15.9%) patients were not tested for puree by reason of severe impairment in the oral bolus control and 29 (46.0%) for the solid consistency due to severe impairment of the oral preparatory swallowing stage, resulting in PAS missing data. Inter-rater agreement proved substantial [46] for both the DOSS and the PAS scales.

Concerning the HRQOL assessment with the I-SWAL-QOL, the 62 participants selected for the criterion validity analysis scored as follows as median (IQR): burden 9.0 (7.0–10.0), Eating duration 7.0 (5.0–9.0), eating desire 14.0 (12.0–15.0), symptoms 55.0 (50.0–62.0), food selection 9.0 (7.0–10.0), communication 6.0 (5.0–8.0), fear 18.5 (16.0–20.0), mental health 22.0 (19.0–25.0), social functioning 24.0 (20.0–25.0), fatigue 11.0 (7.0–13.0), and sleep 8.0 (6.0–10.0).

### Phase 1: I-DHI Development

The final version of the I-DHI is presented as Appendix.

### Phase 2: I-DHI Reliability

Internal consistency and test–retest reliability scores are reported in Table 2. Internal consistency proved satisfactory for all the I-DHI subscales and the Total score with Cronbach’s *α* values ranging from *α* = 0.76 for the Physical subscale to *α* = 0.90 for the Total score. Test–retest reliability proved satisfactory for all I-DHI subscales and Total score, with ICC_agreement_ values exceeding 0.70, ranging from 0.96 for the Physical subscale to 0.99 for the Total score. Similarly, the self-rated OD severity subscale showed adequate test–retest reliability, with weighted Cohen’s *k* = 0.81. Statistically significant differences (*p* < 0.05) were not detected between the main sample and the test–retest subsample compared for age, gender, and diagnosis.

**Table 2:** I-DHI internal consistency and test–retest reliability analyses

I-DHI	Internal consistency (*N* = 75)	Test–retest reliability (*N* = 26)	
	Cronbach’s *α*	ICC_agreement_ (CI)	Weighted Cohen’s *k*
Total	.90	.99 (.98–1.00)	–
Physical	.76	.96 (.90–.98)	–
Functional	.82	.98 (.95–.99)	–
Emotional	.77	.98 (.96–.99)	
OD severity	–	–	.81**

*I-DHI* Italian Dysphagia Handicap Index; *OD* oropharyngeal dysphagia; *ICC*_*agreement*_ intraclass correlation coefficient two-way random effects model, single measures, and absolute agreement; *CI* confidence interval

**p < .01

### Phase 3: I-DHI Validity

#### I-DHI Criterion Validity

Table 3 shows the results of Spearman’s correlation analyses between the I-DHI and the I-SWAL-QoL subscales. Significant inverse moderate to strong correlations were detected between I-DHI and the majority of the I-SWAL-QoL subscales. In particular, the strongest correlations were found between the I-DHI Total score and the I-SWAL-QoL Burden and Social Functioning subscales; between the I-DHI Physical subscale and the I-SWAL-QoL Symptoms subscale; between the I-DHI Functional subscale and the I-SWAL-QoL Eating Duration, Social Functioning, and Fear subscales; between the I-DHI Emotional subscale and the I-SWAL-QoL Social Functioning and Mental Health subscales; between the I-DHI Self-rated OD severity and the I-SWAL-QoL Burden and Symptoms subscales. The weakest and not statistically significant correlations were found for all the I-DHI subscales and Total score with the I-SWAL-QoL Sleep subscale.

**Table 3:**
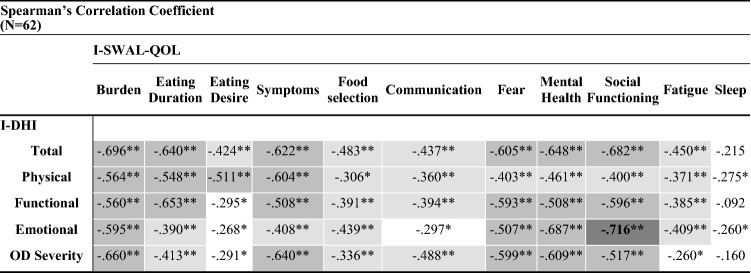
I-DHI criterion validity analysis

Mild, moderate, and strong correlations are reported in light, medium, and dark gray cells

*I-DHI* Italian Dysphagia Handicap Index; *I-SWAL-QOL* Italian-Swallowing Quality of Life Questionnaire; *OD* oropharyngeal dysphagia

*p < .05 (two-tailed); **p < .01 (two-tailed)

#### I-DHI Construct Validity

To investigate construct validity, associations among I-DHI subscales and Total score were assessed with Spearman’s correlation coefficients, as well as associations between I-DHI and OD severity and swallowing safety on FEES. In addition, groups of patients with different OD severity based on either FEES examination or self-assessment were compared using Kruskal–Wallis test.

As presented in Table 4, moderate to strong correlations were found between all the I-DHI subscales and the Total score.

**Table 4:** Correlations among I-DHI subscales

Spearman’s correlation coefficient (*N* = 75)					
	I-DHI				
	Total	Physical	Functional	Emotional	OD severity
*I-DHI*					
Total	–				
Physical	.820**	–			
Functional	.899**	.603**	–		
Emotional	.806**	.528**	.624**	–	
OD severity	.697**	.618**	.607**	.511**	–

*I-DHI* Italian Dysphagia Handicap Index; *OD* oropharyngeal dysphagia

**p < .01 (two-tailed)

A significant moderate inverse correlation was found between I-DHI Total score and OD severity rated through DOSS, while mild inverse correlations between DOSS and the remaining I-DHI subscales were found (Table 5). Only weak significant direct correlations were detected between swallowing safety rated through the worst PAS score and I-DHI Physical subscale, Total, and self-rated OD severity scores. Considering single consistencies separately, I-DHI Physical, Functional, and Total scores were mild to moderate correlated with PAS scores with puree and only mild with liquids. No significant correlation was detected between I-DHI and PAS scores with solids (Table 5).

**Table 5:** Correlations between I-DHI subscales, DOSS, and PAS

Spearman’s correlation coefficient (*N* = 63)					
	DOSS	PAS			
		Worst score	Liquids	Puree	Solids
*I-DHI*					
Total	− .506**	.263*	.360**	.423**	.288
Physical	− .445**	.290*	.321*	.538**	.254
Functional	− .465**	.198	.290*	.336*	.297
Emotional	− .288*	.149	.242	.157	.043
OD severity	− .321*	.258*	.247	.177	.278

*I-DHI* Italian Dysphagia Handicap Index; *OD* oropharyngeal dysphagia; *DOSS* Dysphagia Outcome and Severity Scale; *PAS* Penetration Aspiration Scale

*p < .05 (two-tailed); **p < .01 (two-tailed)

Table 6 shows the comparisons of I-DHI scores among patients with normal swallow (PAS = 1–2), penetration (PAS = 3–5), and aspiration (PAS = 6–8) on FEES, for the worst and each consistency tested. After adjusting *p*-values with Bonferroni’s correction, significantly higher I-DHI Physical and Total scores were detected for patients with aspiration (PAS = 6–8) compared to those with normal swallow (PAS = 1–2), and higher I-DHI Emotional subscale scores compared to penetrators (PAS = 3–5). Considering the single consistencies separately, patients with aspiration of liquids scored higher in I-DHI Physical and Functional subscales and Total score compared to patients with a safer swallow. Patients with aspiration of puree reported higher I-DHI Physical subscale and Total scores compared to those with safe swallow. Patients with aspiration of solids scored higher compared to patients with a safer swallow in all I-DHI subscales, except for the Physical one.

**Table 6:** Comparisons of I-DHI scores among patients (*N* = 63) with normal swallow (PAS = 1–2), penetration (PAS = 3–5), and aspiration (PAS = 6–8) with the worst and each consistency tested

	Worst consistency				Liquids				Puree				Solids			
	PAS 1–2 *N* = 21	PAS 3–5 *N* = 8	PAS 6–8 *N* = 34	Kruskal–Wallis	PAS 1–2 *N* = 30	PAS 3–5 *N* = 12	PAS 6–8 *N* = 21	Kruskal–Wallis	PAS 1–2 *N* = 38	PAS 3–5 N = 7	PAS 6–8 *N* = 8	Kruskal–Wallis	PAS 1–2 *N* = 26	PAS 3–5 *N* = 2	PAS 6–8 *N* = 6	Kruskal–Wallis
*I-DHI*																
Total	20 (12–25)	12 (6.5–30.5)	30 (17.5–50)	**9.453**^**a**^	20 (10–26.5)	18 (6.5–44.5)	32 (22–53)	**11.536**^**c**^	20 (10–28)	30 (22–48)	48 (31–61.5)	**12.516**^**a**^	20 (10–26.5)	6 (2–10)	42 (29.5–56)	**12.578**^**c**^
Physical	6 (4–10)	6 (4–11)	11 (6–18)	**9.024**^**a**^	7 (4–10)	9 (4–15.5)	12 (7–21)	**7.311**^**a**^	6 (4–10)	12 (4–14)	21 (16.5–24)	**17.742**^**a**^	7 (4–10.5)	6 (2–10)	13 (7–24)	4.071
Functional	8 (4–11)	4 (1–17)	11 (4–22)	4.768	7 (4–12.5)	5 (0.5–20)	12 (8–22)	**7.804**^**a**^	8 (4–12)	18 (8–22)	17 (9–26)	7.979	7 (4–12)	0 (0–0)	20 (16–25)	**14.247**^**c**^
Emotional	4 (2–8)	1 (0–4)	7 (2–10.5)	**7.157**^**b**^	4 (2–8)	4 (0–10)	10 (3–12)	5.793	4 (1.5–8.5)	10 (2–10)	10 (0.5–15)	3.097	4 (1.5–8.5)	0 (0–0)	9 (3.5–12)	**6.497**^**b**^
OD severity	3 (2–4)	2.5 (1–4)	4 (2.8–4)	5.445	3 (2–4)	3.5 (1–4)	4 (3–5)	6.426	3 (2–4)	4 (2–4)	4 (2.3–4.8)	2.014	3 (2–4)	2 (1–3)	4.5 (3.8–6)	**7.621**^**a**^

Italian Dysphagia Handicap Index (I-DHI) scores are presented as median (IQR). Significant comparisons after Bonferroni’s correction are reported in bold

*OD* oropharyngeal dysphagia; *PAS* Penetration Aspiration Scale

^a^Significant differences (*p* < .05; two-tailed) between patients with normal swallow (PAS = 1–2) and aspirators (PAS = 6–8)

^b^Significant differences (*p* < .05; two-tailed) between penetrators (PAS = 3–5) and aspirators (PAS = 6–8)

^c^Significant differences *(p* < .05; two-tailed) between aspirators (PAS = 6–8) and either patients with normal swallow (PAS = 1–2) or penetrators (PAS = 3–5)

**Fig. 1:**
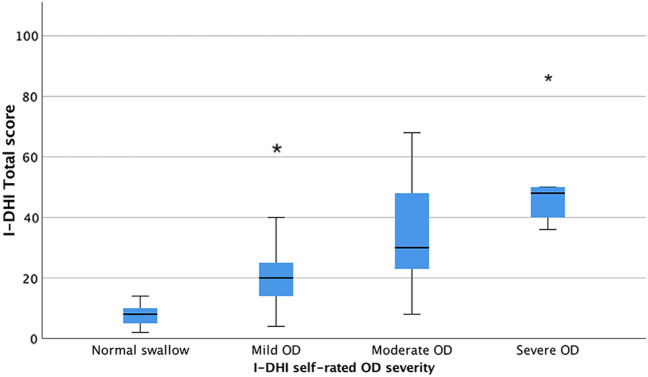
I-DHI Total score distribution for the self-rated OD severity groups

Figure 1 shows the distribution as box plots of I-DHI Total score among patients with different self-rated OD severity (normal swallow = 1; mild swallowing problem = 2–3; moderate swallowing problem = 4–5; severe swallowing problem = 6–7). Significantly higher I-DHI Total scores were reported by patients with more severe perceived OD among all severity groups (Kruskal–Wallis 36.648; *p* < .05; median (IQR) of normal swallow 8 (4–10), mild OD 20 (14–26.5), moderate OD 30 (22–50), and severe OD 48 (38–68) groups). Only patients with moderate and severe perceived OD did not differ significantly in the I-DHI Total scores. A similar trend was observed when considering separately each I-DHI subscale, although significance was not reached for all group comparisons. Table 7 presents the comparisons between I-DHI scores for each self-rated OD severity group.

**Table 7:** Comparisons of I-DHI subscales scores among patients with different self-rated OD severities

	I-DHI self-rated OD severity				
	Normal swallow *N* = 11	Mild *N* = 24	Moderate *N* = 35	Severe *N* = 5	Kruskal–Wallis
*I-DHI*					
Physical	4 (2–6)	6 (4–10)	14 (10–18)	10 (7–19)	**28.821**^**a**^
Functional	2 (0–4)	8 (4–12)	12 (8–22)	24 (16–30)	**28.597**^**b**^
Emotional	0 (0–4)	4 (2–8)	8 (2–10)	16 (12–21)	**25.214**^**b**^

Italian Dysphagia Handicap Index (I-DHI) scores are presented as median (IQR). Significant comparisons after Bonferroni’s correction are reported in bold

*OD* oropharyngeal dysphagia

^a^Significant differences (*p* < .05; two-tailed) between moderate and either normal swallow or mild OD groups

^b^Significant differences (*p* < .05; two-tailed) between normal swallow and either moderate or severe OD groups and between mild and severe OD groups

Concerning clinical validity, OD patients reported significantly higher scores in all the I-DHI subscales and the Total score when compared to healthy participants with the U Mann–Whitney test (*p* < .05) (Table 8).

**Table 8:** Comparisons of I-DHI scores between patients with OD and healthy participants

	Patients with OD *N* = 75	Healthy participants *N* = 166	*U* Mann–Whitney	*z* score	*p* (two-tailed)
*I-DHI*					
Total	22 (14–40)	2 (0–4)	274.000	− 12.087	** < .001**
Physical	10 (5–14)	2 (0–4)	928.500	− 10.812	** < .001**
Functional	10 (4–18)	0 (0–0)	844.000	− 13.601	** < .001**
Emotional	4 (2–10)	0 (0–0)	1382.000	− 12.514	** < .001**
OD severity	4 (2–4)	1 (1–1)	959.500	− 13.329	** < .001**

Italian Dysphagia Handicap Index (I-DHI) scores are presented as median (IQR). Significant *p* are reported in bold

*OD* oropharyngeal dysphagia

### Phase 4: I-DHI Interpretability

The features of I-DHI distribution are reported in Table 9. For each I-DHI subscale the number of items, the number of observed different scores, the possible and observed score range, the median (IQR), and the percentage of participants who reported the lowest and highest possible scores are described. A floor effect was detected for the Emotional subscale only. A trend toward floor effect was observed for self-rated OD severity and the Functional subscale.

**Table 9:** Features of I-DHI distribution

I-DHI	N. items	N. distinct scores	Possible range	Observed range	Median (IQR)	% Floor	% Ceiling
Total	25	30	0–100	2–86	22 (14–40)	0	0
Physical	9	13	0–36	2–26	10 (4–14)	0	0
Functional	9	17	0–36	0–36	10 (4–18)	13.3	1.3
Emotional	7	12	0–28	0–24	4 (2–10)	21.3*	0
OD severity	1	7	1–7	1–7	4 (2–4)	14.7	1.3

*I-DHI* Italian Dysphagia Handicap Index; *OD* oropharyngeal dysphagia

*Floor effect present (more than 15% of respondents achieved the lowest possible score)

I-DHI scores were compared among gender and age (18–39, 40–64, ≥ 65 years) groups of patients and healthy participants using U Mann–Whitney and Kruskal–Wallis tests, respectively. As regards patients, no significant differences (*p* < .05) were detected. Healthy participants reported 2 (0–4) as median (IQR) I-DHI Total score, 2 (0–4) in the Physical, 0 (0–0) in the Functional, and 0 (0–0) in the Emotional subscales. Concerning healthy participants, no significant differences were detected for gender. Conversely, individuals aged ≥ 65 years reported significantly higher (*p* < .05) I-DHI Physical subscale and Total scores. Norm values for age class are presented in Table 10.

**Table 10:** I-DHI norm values

	Healthy participants			
	18–39 years *N* = 65	40–64 years *N* = 72	≥ 65 years *N* = 27	Kruskal–Wallis
*I-DHI*				
Total	2 (0–2)	2 (0–4)	4 (2–4)	**8.089**^**a**^
Physical	2 (0–2)	2 (0–4)	4 (2–4)	**9.046**^**a**^
Functional	0 (0–0)	0 (0–0)	0 (0–0)	0.000
Emotional	0 (0–0)	0 (0–0)	0 (0–0)	1.789
OD severity	1 (1–1)	1 (1–1)	1 (1–1)	2.189

Italian Dysphagia Handicap Index (I-DHI) scores are presented as median (IQR). Significant comparisons are reported in bold

*OD* oropharyngeal dysphagia

^a^Significant differences (*p* < .05; two-tailed) between ≥ 65 years and either 18–39 or 40–64 years groups

## Discussion

The DHI is a PRO measure of HRQOL for adults with OD, developed for English-speaking populations and to date adapted into Arabic [30], Persian [31, 32], Japanese [33], Hebrew [34], Kannada [35], and Korean [36]. In compliance with these previous validation studies, the I-DHI resulted in a 25-item tool, easy to apply in clinical practice since all the participants managed to complete the questionnaire autonomously in no more than 10 min.

I-DHI psychometric properties were examined in a sample of 75 patients with OD of etiological heterogeneity and 166 healthy participants. Patients’ etiologies were predominantly, but not limited to, chronic conditions such as neurodegenerative disorders and head and neck cancer (HNC), as they represent the most prevalent populations in the authors’ clinical practice. The high prevalence of chronic conditions may be seen as a strength of the present study. In fact, the different extent of OD impact on HRQOL might be more evident in patients affected by chronic rather than acute diseases, as they adapted to a stable or slowly progressing condition.

Concerning reliability analyses, similarly to the previous DHI validation studies [11, 30–35], the I-DHI internal consistency met satisfactory parameters (Cronbach’s *α* = .70–.90). In particular, *α* values between 0.76 and 0.82 were detected for the I-DHI subscales and an *α* value equal to 0.90 for the I-DHI Total score. This suggests a strong interrelatedness between the items of the questionnaire and of each subscale. Alpha values lower than 0.70 reflect a low correlation among the instrument items and may suggest an insufficient and/or poorly chosen set of items. Conversely, *α* values higher than 0.90 reflect a high correlation among the instrument items and may suggest redundancy of items [50]. The obtained *α* values could be interpreted as satisfactory I-DHI internal consistency, and thus no I-DHI items were inserted nor deleted. I-DHI test–retest reliability proved satisfactory, thus confirming I-DHI high stability and reliability over time.

In the present study, I-DHI validity was established performing criterion and construct validity analyses. The assessment of I-DHI criterion validity represents a novelty, since it had never been performed by previous DHI validation studies. To establish I-DHI criterion validity the Italian version of the SWAL-QoL was chosen as the gold standard. Mild to strong inverse correlations between I-DHI and I-SWAL-QoL subscales were found. As could be logically expected, the lowest correlations (*ρ* < .30) were detected for the Communication, Fatigue, and Sleep I-SWAL-QoL subscales since they investigate domains which are not included in the I-DHI. Satisfactory criterion validity is defined by Terwee et al. [48] as the presence of correlations with a gold standard > .70 in absolute value. This condition was verified only for the correlation between the I-DHI Emotional subscale and the Social Functioning subscale of the I-SWAL-QoL. Nevertheless, the correlations between the remaining I-DHI and I-SWAL-QoL subscales show a trend toward this cut-off level.

I-DHI construct validity proved satisfactory, since the results confirmed > 75% of the hypotheses made by the authors [48]. In particular, in accordance with the results of the previous validation studies [11, 30–35], direct moderate to strong (> .50–.70) correlations among I-DHI subscales, Total score, and self-rated OD severity were found. Nevertheless, due to the insufficient numerousness of the recruited sample, factor analysis was not performed in the present study nor in any of the previous DHI validations. In fact, 4–10 participants per item within a sample composed at least of 100 participants are necessary to perform factor analyses [48, 50]. As such, no claims can be made as to the number and reciprocal relations of the construct(s) investigated through the I-DHI. Consistently with authors’ hypotheses and with the Hebrew validation study [34], I-DHI was mild to moderate associated with OD severity measured on FEES and only weakly with swallowing safety. Among the previous DHI validation studies, the English [11], Japanese [33], Hebrew [34] Kannada [35], and Korean [36] investigated the possible relations between DHI and instrumental assessment of OD. All the aforementioned studies used VFSS for the assessment of swallowing performance, with the unique exception of Shapira-Galitz et al. [34], which performed FEES examinations scoring swallowing safety with PAS. They found significant differences in the DHI Functional subscale scores only between non-aspirators (PAS = 1) and patients with penetration or aspiration of any degree (PAS ≥ 2). These findings partially overlap with the ones of the present study. However, in the present study patients were considered as having a safe swallow for PAS scores = 1–2 [44, 45] and analyses were performed on individual consistencies in addition to the worst PAS score. Differently from Shapira-Galitz et al. [34], comparing groups of patients with different swallowing safety, higher I-DHI Total scores were detected for patients with aspiration compared to those with a safer swallow for all the consistencies tested. A similar trend was detected among all I-DHI subscales, although with differences depending on the consistency. Nevertheless, caution should be paid when interpreting these results with both puree and solid consistencies owing to the limited number of patients in the penetration and aspiration groups compared to the normal swallow group.

In line with the hypotheses and previous validation studies [11, 30–36], higher I-DHI Total scores (meaning worse HRQOL) were reported by patients with more severe perceived OD for all group comparisons, except for the moderate and severe perceived OD groups. On examining I-DHI subscales separately, a similar trend was identified, although not significant for all group comparisons. This might be explained by the unequal number of patients in each group and does not necessarily imply the absence of a real difference. In fact, in the present study only 5 patients perceived severe OD.

All the DHI validation studies [11, 30–36], except for the DHI Persian one [31, 32], investigated clinical validity reporting satisfactory findings. Similarly, the significantly higher I-DHI scores reported by OD patients compared to healthy participants adequately reflect the impact of OD on patients’ HRQOL. In addition, Sobol et al. [51] developed DHI normative values through a systematic review and meta-analysis, reporting 2.49 (0.51–4.48) as DHI mean (CI). The normative data generated in the present study aligns with Sobol et al. [51].

As pertains to I-DHI interpretability, the sample included in the present study reported overall moderate to high levels of HRQOL (i.e., low I-DHI scores) for each I-DHI subscale and the Total score. Similarly, high levels of HRQOL were on investigating HRQOL with the I-SWAL-QoL. Patients included in the present study perceived a normal or slightly impaired swallowing function, while on FEES examination at least 50% of them showed moderate to severe OD, based on DOSS scores. Thus, a discrepancy between instrumental and self-assessed OD severity may be suggested, as reported by Speyer et al. [8]. A possible explanation for this discrepancy and the low I-DHI scores reported in the present study could be the high prevalence of chronic conditions within the recruited sample, as patients might have already found strategies to adapt to OD. This hypothesis might be supported by the floor effect detected for the I-DHI Emotional subscale and the trend toward floor effect for the Functional and self-rated OD severity subscales, but not for the Physical one. The I-DHI median values reported in the present study overlaps with the English validation findings [11], while all the other DHI validation studies [30, 33–35] detected overall higher scores. As reported by Krishnamurthy et al. [35], the DHI different range scores obtained in each validation may be explained by differences in the samples OD severity, both instrumentally assessed and self-rated.

Finally, no differences in I-DHI scores were found comparing patients with different gender and age, suggesting that I-DHI can be scored without correcting for these demographic characteristics. Older healthy participants (≥ 65 years), instead, presented higher I-DHI scores compared to younger ones. This could be explained by the physiological deterioration of swallowing function in older people [52].

## Limitations and Future Directions

The present study is not exempt from limitations. In fact, the relatively small sample size recruited warns caution when interpreting the present results, although they are fundamentally comparable to previous DHI validations. In addition, the modest heterogeneity of OD etiologies of the recruited sample could be considered as a limitation of the present study. Nevertheless, the vast majority of the recruited patients suffered from chronic conditions, which may be seen as a strength for the investigation of OD impact on HRQOL. Conversely, future studies aiming at analyzing I-DHI responsiveness could benefit from recruiting patients affected by acute conditions, since changes in HRQOL and thus in I-DHI scores may be more evident in these populations. For safety reasons, FEES missing data mainly referred to puree and solid consistencies. Thus, the interpretation of the comparisons of patients who differed for swallowing safety on FEES with different consistencies requires caution.

Finally, the absence of factor and responsiveness analyses should be acknowledged as a limitation of the present study, since based on the present results, no claims can be made about the construct(s) underlying I-DHI and I-DHI ability to report clinically significant changes over time.

Future research studies on larger sample size of patients with equally represented acute and chronic conditions besides more heterogeneous OD severity are required in order to confirm the results of the present study. Furthermore, the number of constructs underlying the I-DHI and I-DHI ability to detect clinically significant changes in OD patients’ HRQOL should be investigated with factor and responsiveness analyses. In the present study I-DHI psychometric properties were investigated based on Classical Test Theory (CTT). Together with Item Response Theory (IRT), CTT is a measurement theory necessary for measuring an unobservable construct (e.g., OD patients’ HRQOL) by means of its manifestations (e.g., questionnaire items). Using CTT, conclusions on the quality of the measurement properties of an instrument are drawn at the test level and are population-dependent. Conversely, IRT examines the contribution of each item to the construct under measure, allowing conclusions independently from the tested population, yet it requires a relatively large sample size and stronger assumptions on the data to fit the chosen IRT model. Future studies may add information on I-DHI psychometric properties based on IRT using Rasch Analysis techniques.

## Conclusion

I-DHI is a reliable, valid, easy to administer, and symptom-specific patient-reported outcome to investigate HRQOL in Italian adults with OD. I-DHI showed overall satisfactory reliability and construct validity, while criterion validity with I-SWAL-QoL as the gold standard seems promising. The presentation of score distribution and norm values fosters I-DHI interpretability. Factor and responsiveness analyses should be performed in future studies.

## Appendix

Final version of the I-DHI. Italian-translated items are reported in italics.

**Dysphagia Handicap Index (DHI)**

*Italian Dysphagia Handicap Index (I-DHI)*

Please place a check in the box that describes your swallowing difficulty.

*Per favore metta un segno nella casella che corrisponde alla sua difficoltà deglutitoria*.

**Table:**

		Never *Mai*	Sometimes *Qualche volta*	Always *Sempre*
1P	I cough when I drink liquids *Tossisco quando bevo i liquidi*			
2P	I cough when I eat solid food *Tossisco quando mangio i cibi solidi*			
3P	My mouth is dry *La mia bocca è secca*			
4P	I need to drink fluids to wash food down *Ho bisogno di bere liquidi per far scendere il cibo*			
5P	I’ve lost weight because of my swallowing problem *Ho perso peso a causa del mio problema di deglutizione*			
1F	I avoid some foods because of my swallowing problem *Evito alcuni cibi a causa del mio problema di deglutizione*			
2F	I have changed the way I swallow to make it easier to eat *Ho cambiato il modo in cui deglutisco per mangiare più facilmente*			
1E	I’m embarrassed to eat in public *Sono imbarazzato a mangiare in pubblico*			
3F	It takes me longer to eat a meal than it used to *Impiego più tempo a consumare un pasto di quanto fossi abituato*			
4F	I eat smaller meals more often due to my swallowing problem *Consumo pasti più piccoli, più frequentemente a causa del mio problema di deglutizione*			
6P	I have to swallow again before food will go down *Devo deglutire di nuovo prima che il cibo scenda*			
2E	I feel depressed because I can’t eat what I want *Mi sento depresso perché non posso mangiare quello che voglio*			
3E	I don’t enjoy eating as much as I used to *Non provo piacere nel mangiare come ero abituato*			
5F	I don’t socialize as much due to my swallowing problem *Non socializzo così tanto a causa del mio problema di deglutizione*			
6F	I avoid eating because of my swallowing problem *Evito di mangiare a causa del mio problema di deglutizione*			
7F	I eat less because of my swallowing problem *Mangio di meno a causa del mio problema di deglutizione*			
4E	I am nervous because of my swallowing problem *Sono nervoso a causa del mio problema di deglutizione*			
5E	I feel handicapped because of my swallowing problem *Mi sento handicappato a causa del mio problema di deglutizione*			
6E	I get angry at myself because of my swallowing problem *Mi arrabbio con me stesso a causa del mio problema di deglutizione*			
7P	I choke when I take my medication *Mi soffoco quando prendo le mie medicine*			
7E	I’m afraid that I’ll choke and stop breathing because of my swallowing problem *Ho paura di soffocare e smettere di respirare a causa del mio problema di deglutizione*			
8F	I must eat another way (e.g., feeding tube) because of my swallowing problem *Devo mangiare in un altro modo (es. sondino naso gastrico) a causa del mio problema di deglutizione*			
9F	I’ve changed my diet due to my swallowing problem *Ho cambiato la mia dieta a causa del mio problema di deglutizione*			
8P	I feel a strangling sensation when I swallow *Sento una sensazione di strangolamento quando deglutisco*			
9P	I cough up food after I swallow *Tossisco fuori il cibo dopo averlo deglutito*			

Please circle the number that matches the severity of your swallowing difficulty (1 = no difficulty at all; 4 = somewhat of a problem; 7 = the worse problem you could have) *Per favore cerchi il numero che corrisponde alla gravità della sua difficoltà di deglutizione* *(1* = *nessuna difficoltà affatto; 4* = *un po’ un problema; 7* = *il problema peggiore che si possa avere)*						
1	2	3	4	5	6	7
Normal *Normale*			Moderate problem *Problema moderato*			Severe problem *Problema grave*
